# Delayed hepatic response and impaired cytokine dynamics in aged mice following burn injury: Implications for elderly patient care

**DOI:** 10.1371/journal.pone.0316813

**Published:** 2025-02-24

**Authors:** Israel Muro, Andrea C. Qualman, Kenneth Meza Monge, Akshay Pratap, Elizabeth J. Kovacs, Juan-Pablo Idrovo

**Affiliations:** 1 Department of Surgery, Division of G.I., Trauma, and Endocrine Surgery, University of Colorado, Aurora, Colorado, United States of America; 2 Department of Immunology and Microbiology, University of Colorado, Aurora, Colorado, United States of America; Pacific Northwest National Laboratory, UNITED STATES OF AMERICA

## Abstract

**Introduction:**

Burn injuries in elderly patients result in higher morbidity and mortality compared to younger individuals. This study investigates age-related differences in inflammatory hepatic responses to burn injuries.

**Method:**

Young (8–10 weeks) and aged (20-21 months) female C57BL/6 mice were subjected to a 15% total body surface area burn or sham injury. Serum and liver samples collected at 3, 6-, 9-, 12-, and 24-hours post-injury were analyzed for serum amyloid A (SAA) levels, SAA1 and SAA2 hepatic gene expression, serum cytokines (IL-6, IL-1β, TNF-α, and IL-10), and hepatic STAT3 activation.

**Results:**

Aged mice showed a delayed and dysregulated response. In young mice, SAA levels rose significantly at 6 hours postburn (5.09 ±  0.2-fold), while in aged mice, SAA increased at 12 hours (39.1 ±  2.06-fold), p <  0.01. Hepatic expression of SAA1 and SAA2 also peaked early in young mice (8.357 ±  1.257-fold and 5.91 ±  0.664-fold at 3 hours) but was delayed until 12 hours in aged mice. Young mice demonstrated early IL-6 peaks at 3 hours (990 ±  83.2 pg/ml), while aged mice reached a delayed, higher IL-6 peak at 24 hours (3804 ±  1408 pg/ml, p <  0.05). Similar age-related delays occurred for IL-1β and TNF-α. Aged mice had significantly elevated IL-10 at 6 hours (993.9 ±  99.41 pg/ml vs. 67.69 ±  6.635 pg/ml in young, p <  0.001). STAT3 activation peaked at 3 hours in young mice (2.686 ±  0.226-fold) but was delayed until 24 hours in aged mice (0.5958 ±  0.0368-fold, p <  0.05).

**Conclusions:**

This study identifies age-related variations in inflammatory markers and acute hepatic responses to burn injuries, with aged mice showing delayed and reduced inflammatory responses compared to younger counterparts. These findings underscore the importance of age-specific strategies in burn injury management to enhance outcomes for elderly burn patients.

## Introduction

Burn injuries in elderly patients present a significant clinical challenge due to markedly higher rates of morbidity and mortality compared to younger individuals [1–3]. Burn injuries initiate a complex systemic inflammatory response, which impacts multiple organ systems and can lead to complications such as infection, sepsis, and multi-organ failure. This response, known as “systemic inflammatory response syndrome” (SIRS), triggers metabolic shifts, immune activation, and fluid imbalances, all of which increase the risk of poor outcomes following burn injuries [4,5]. In younger individuals, the body’s response to these challenges tends to be more resilient and coordinated, allowing for a quicker and more effective recovery [6]. However, in elderly patients, the physiological response to burn injuries is often compromised, leading to a delayed and less effective recovery process [7]. Aging affects various organs’ ability to respond to and recover from trauma, with the liver—a central organ in metabolic and immune responses—being significantly impacted [8].

The liver plays a vital role in managing the systemic stress response post-burn, mainly through the production of acute phase proteins (APPs) like serum amyloid A (SAA), C-reactive protein (CRP), and fibrinogen, which are crucial for modulating inflammation and facilitating tissue repair [8]. Following burn injuries, immune cells such as macrophages and neutrophils are activated, producing a range of cytokines that enter the bloodstream, traveling to target organs. These cytokines, including interleukin-6 (IL-6), interleukin-1 beta (IL-1β), tumor necrosis factor-alpha (TNF-α), and interleukin-10 (IL-10), reach the liver, where they act as signaling molecules to activate hepatic cells, initiating the acute phase response [9,10]. This communication between immune cells and the liver is essential for coordinating a systemic inflammatory response that supports tissue repair and defends against infection [8].

IL-6 is a primary activator of the hepatic acute phase response, where it binds to receptors on hepatocytes and triggers the activation of the Signal transducer and activator of transcription 3 (STAT3) signaling pathway. This activation leads to the transcription of genes encoding APPs, such as SAA and CRP, which play crucial roles in the inflammatory response, wound healing, and immune modulation [11]. The rapid upregulation of IL-6 and subsequent STAT3 activation help the liver to respond promptly to injury, stabilizing systemic inflammation. However, persistent elevation of IL-6, which may occur in severe or poorly controlled inflammation cases, can lead to adverse effects, including liver dysfunction and even fibrosis [12].

TNF-α, another central cytokine, initiates the systemic inflammatory response and plays a complex role in hepatic metabolism and immune function. TNF-α promotes hepatocyte apoptosis and the release of other inflammatory mediators. When dysregulated, it can contribute to hepatocellular damage and disrupt metabolic pathways necessary for recovery post-injury [13]. TNF-α also stimulates the production of reactive oxygen species (ROS), which can further damage liver cells and impair their regenerative capacity following a burn injury. In the context of aging, TNF-α responses are often delayed or dysregulated, exacerbating inflammatory liver damage in elderly burn patients [14].

IL-1β acts synergistically with IL-6 and TNF-α in amplifying inflammatory responses within the liver through activation of the nuclear factor kappa B (NF-κB) pathway. NF-κB signaling promotes the expression of pro-inflammatory genes, enhancing cytokine and chemokine production and recruiting immune cells to the liver [15]. This pathway is crucial in the initial inflammatory response; however, prolonged or excessive IL-1β activity can lead to chronic liver inflammation and impair liver function. In aged individuals, IL-1β responses may be altered, leading to inadequate or delayed initiation of the acute phase response and compromising the liver’s ability to regulate systemic inflammation effectively [16].

IL-10, an anti-inflammatory cytokine, plays a regulatory role by modulating the liver’s response to pro-inflammatory signals. It suppresses the synthesis and signaling of IL-6, IL-1β, and TNF-α, acting as a critical counterbalance to prevent excessive inflammation. By limiting the pro-inflammatory cytokine response, IL-10 helps mitigate hepatic tissue damage, preserve liver function, and promote the resolution of inflammation. However, in elderly individuals, the regulation and timing of IL-10 production can be compromised, potentially leading to prolonged inflammation that exacerbates tissue injury and delays recovery [17].

Although women have been shown to have worse outcomes than men after burn injuries, particularly in older age groups [18,19], the underlying mechanisms of this sex difference are beyond the scope of this study. Nonetheless, these observations highlight the importance of considering sex and age as relevant biological factors in burn research.

The high mortality and morbidity seen in elderly burn victims emphasize the need for studies that specifically examine age-related physiological differences in response to trauma. Previous research has shown that older individuals experience a delayed or diminished acute phase response, but there is limited information on how this affects the hepatic response, specifically in the context of burn injuries [6,7,20]. By comparing young and aged mice, this study seeks to clarify how aging alters the liver’s ability to respond to burn-induced stress. Analyzing age-related differences in hepatic function, APP production, and cytokine levels within the critical early hours post-burn could provide essential insights. Ultimately, findings from studies like this can help guide age-specific therapeutic strategies that improve outcomes and quality of care for elderly burn victims, who are particularly vulnerable to complications due to age-related declines in physiological resilience.

## Materials and methods

### Mice

Female C57BL/6 mice, both young (8–10 weeks old, comparable to 20–25 human years) and aged (20–21 months old, equivalent to 65–70 human years), were procured from The Jackson Laboratory (Bar Harbor, ME, USA) and the National Institute of Aging (NIA) Colony (Charles River Laboratories, Wilmington, MA, USA), respectively. Before experiments began, the mice were acclimatized at the University of Colorado Anschutz Medical Campus Vivarium for at least two weeks. Burn injuries took place between 9 and 11 am to reduce the impact of circadian rhythms. All efforts were made to minimize animal suffering and distress. Mice were monitored regularly for signs of pain or distress, with particular attention during the post-burn period. Euthanasia was performed using gradual CO2 exposure followed by cervical dislocation under the University of Colorado Institutional Animal Care and Use Committee (IACUC) guidelines (IACUC protocol no. 00001163).

### Burn injury model

The mice were randomly divided into four experimental groups: young sham, young burn, aged sham, and aged burn, with 10 mice per group per timepoint. A 15% total body surface area (TBSA) full-thickness burn was induced under anesthesia using 55.5 mg/kg of ketamine and 2.6 mg/kg of xylazine (Webster Veterinary, Sterling, MA). As previously described [21–24], the dorsum of each mouse was shaved, and templates were used to expose the correct amount of skin based on the mouse’s mass [25]. Burn injuries were induced by immersing the exposed skin in 95°C water for 10 seconds. Sham groups underwent the same procedure using room-temperature water (25°C) for the same duration. Considering the approximately 10-hour half-life of buprenorphine extended-release (ER) and the study’s duration, mice received a single intraperitoneal injection of 0.13 mg/kg buprenorphine ER for pain relief, along with 1 mL of normal saline for fluid resuscitation after treatment. Blood samples were collected immediately after euthanasia at 3, 6-, 9-, 12-, and 24 hours post-burn and centrifuged at 10,000 ×  g for 5 minutes at 4°C to isolate serum. Livers were harvested at the same timepoints, snap-frozen in liquid nitrogen, and stored at –80°C. Homogenized liver tissues were kept on ice until analysis.

### Serum amyloid enzyme-linked immunosorbent assay (ELISA)

Serum levels of Serum Amyloid A1 and A2 (Saa1 and Saa2) were measured using the Mouse Serum Amyloid A DuoSet ELISA kit (R&D Systems, no. DY2948-05). Due to the high homology (91%) between Saa1 and Saa2, the ELISA antibodies detect both proteins simultaneously. The assay was performed following the manufacturer’s instructions.

### Quantitative polymerase chain reaction (qPCR)

RNA extraction from liver tissue was carried out using the RNeasy Mini Kit (no. 74106, Qiagen). The iScript cDNA Synthesis Kit (no. 1708891, BioRad) was employed to synthesize cDNA [22,23,26]. TaqMan probes and TaqMan Universal PCR Master Mix (no. 4304437) were obtained from Fisher Scientific for qPCR analysis of Saa1 (no. Mm00656927) and Saa2 (no. Mm04208126). GAPDH served as the endogenous control (no. 4352339E, Fisher Scientific).

### Serum cytokine analysis

The serum was separated from collected blood by centrifugation at 10,000 ×  g for 10 minutes at 4°C. According to the manufacturer’s protocol, the serum levels of cytokines IL-6, IL-1β, TNF-α, and IL-10 were quantified using the V-PLEX Pro-inflammatory Panel 1 Mouse kit (#N05048A-1, Rockville, MD). Concentrations were calculated using the MSD Discovery Workbench Software, based on a standard curve using a four-parameter logistic model with 1/y^2^ weighting [27,28].

### Western blotting

Liver tissues were homogenized and sonicated in a lysis buffer containing 20 mM Tris (pH 7.4), 150 mM sodium chloride (NaCl), 1 mM ethylenediaminetetraacetic acid (EDTA), 1 mM EGTA, 1% Triton, 0.1% sodium dodecyl sulfate (SDS), and a protease inhibitor cocktail [29]. Protein concentrations were measured using the Protein Assay Dye Reagent (no. 5000006, BioRad). Twenty micrograms of protein were loaded onto polyacrylamide gels (no. 4568085, BioRad) for electrophoresis. Proteins were transferred to membranes using the Nitrocellulose Transfer Kit (no. 1704270; Bio-Rad) and a Trans-Blot Turbo Transfer System (#1704150; Bio-Rad). Membranes were blocked with blotting buffer (no. 12010020, Bio-Rad) and incubated with primary antibodies overnight at 4°C in 1X TBS (no. 1706435, BioRad) containing 0.1% Tween-20 (Sigma, P1379) and 2% bovine serum albumin (BSA; no. B2518, Sigma). The primary antibodies used were recombinant anti-STAT3 (phospho Y705) antibody [EP2147Y] (ab76315) and recombinant anti-STAT3 antibody [EPR787Y] (ab68153). Horseradish peroxidase (HRP)-linked anti-rabbit IgG was used as the secondary antibody (Cell Signaling #7074). Detection was performed using ECL Western Blotting Substrate (no. 32106, Fisher Scientific), and blots were visualized on a ChemiDoc Imaging System (no. 12003153, Bio-Rad). Densitometric analysis was done using Image Lab 6.1, normalizing total STAT3 to phospho-STAT3.

### Statistical analysis

Power analysis was conducted using software from the Colorado Biostatistics Consortium, part of the Department of Biostatistics and Informatics at the Colorado School of Public Health, University of Colorado, Denver, USA. The sample size (n =  10 per group per timepoint) was determined based on previous studies using this model and the significant outcome differences between young and aged mice [22,30,31]. Data were analyzed with GraphPad Prism 10.2.3 software. One-way ANOVA followed by Tukey’s post hoc test was used for statistical comparisons between groups, with significance set at p <  0.05 (indicated by * ). Distribution normality was assessed using column statistics, the Shapiro-Wilk Test, and Q-Q plots in GraphPad Prism. The homogeneity of variance was evaluated using the Brown–Forsythe test.

The ARRIVE guidelines were followed to ensure accurate and thorough reporting of the methods, results, and discussions.

## Results

### Delayed hepatic response in aged mice following burn injury

**Fig 1:**
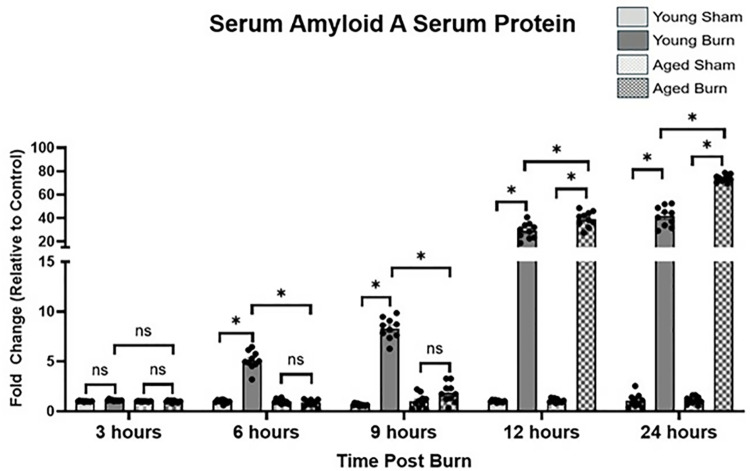
Serum amyloid A (SAA) protein levels in serum of young and aged mice following burn injury measured by ELISA. Data are presented as fold change relative to the age-matched sham group (±SEM). n = 10 per group. * p < 0.05, One-way ANOVA. Tukey’s multiple comparisons.

SAA, a sensitive marker of the acute phase response and a crucial indicator of hepatic function during inflammation [14], was measured to assess the liver’s response to burn injury. Significant age-related differences emerged in the acute phase response pattern when comparing young and aged burn mice. At 3 hours post-burn, SAA in young and aged burn mice showed minimal elevation with no significant differences among the study groups. By 6 hours, young burn mice exhibited significantly higher SAA levels (5.095 ±  0.288-fold) compared to aged burn mice (0.841 ±  0.103-fold, p <  0.05). This difference persisted at 9 hours, with young burn mice maintaining higher levels (8.299 ±  0.338-fold) compared to aged burn mice (1.839 ±  0.304-fold, p <  0.05). At 12 hours, both groups showed substantial increases, with aged burn mice (39.19 ±  2.067-fold) exhibiting significantly higher levels than young burn mice (29.06 ±  2.105-fold, p <  0.05). This enhanced response in aged mice continued throughout 24 hours (74.04 ±  0.973-fold vs. 41.47 ±  2.659-fold in young burn mice, p <  0.05). (Fig 1).

### Hepatic gene expression of acute phase proteins

To understand the transcriptional regulation underlying the SAA protein response, we analyzed the expression of SAA1 and SAA2 genes, which encode the major acute-phase proteins produced by hepatocytes in response to inflammatory stimuli [32]. Analysis of SAA1 gene expression revealed distinct temporal patterns between age groups. At 3 hours, young burn mice showed significantly higher expression (8.357 ±  1.257-fold) compared to aged burn mice (1.466 ±  0.229-fold, p <  0.05). This difference was more pronounced at 6 hours, with young burn mice exhibiting markedly higher expression (155.8 ±  27.7-fold) compared to aged burn mice (0.734 ±  0.180-fold, p <  0.05). The most striking difference occurred at 9 hours, where young burn mice showed dramatically higher expression (6393 ±  989.8-fold) compared to aged burn mice (65.94 ±  15.25-fold, p <  0.05). However, by 12 hours, aged burn mice demonstrated comparable expression levels (831.0 ±  68.99-fold vs. 700.2 ±  109.9-fold in young burn mice, not significant), with similar levels maintained through 24 hours (419.9 ±  45.24-fold in aged vs. 370.2 ±  35.37-fold in young, not significant).

**Fig 2:**
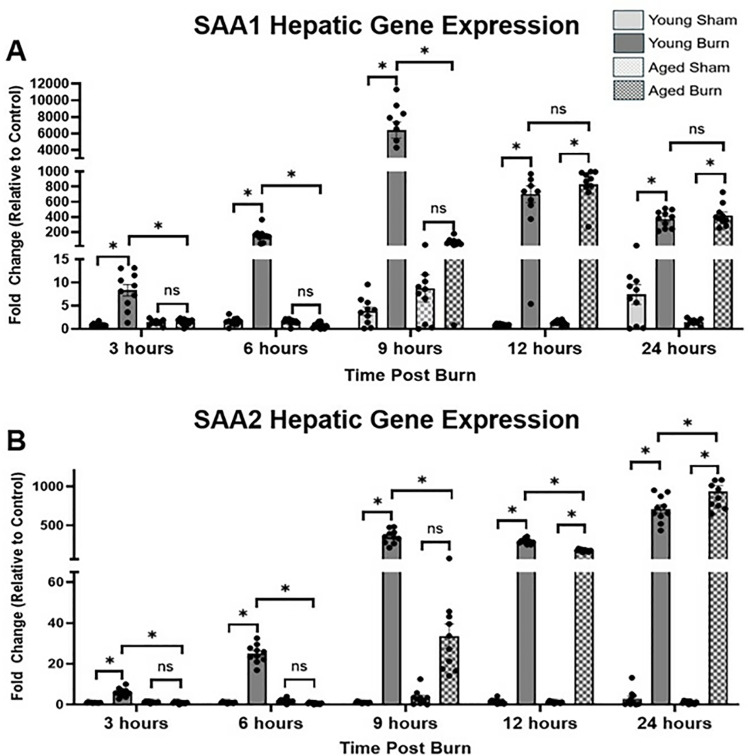
Hepatic gene expression of SAA1 and SAA2 in young and aged mice post-burn. Gene expressions in whole livers were analyzed using qPCR. (A) SAA1 (B) SAA2. Results were determined using the ΔΔCt algorithm with GAPDH as an internal control. Data are presented fold change relative to the age-matched sham group (±SEM). n = 10 per group. * p < 0.05, One-way ANOVA. Tukey’s multiple comparisons.

SAA2, which works in concert with SAA1 to mount an effective acute phase response [33], followed similar age-dependent patterns. At 3 hours, young burn mice exhibited significantly higher expression (5.91 ±  0.664-fold) compared to aged burn mice (0.984 ±  0.138-fold, p <  0.05). This difference increased at 6 hours, with young burn mice showing higher expression (24.98 ±  1.429-fold) compared to aged burn mice (0.722 ±  0.126-fold, p <  0.05). At 9 hours, the difference remained significant, with young burn mice exhibiting higher expression (355.3 ±  27.54-fold vs. 33.61 ±  6.041-fold in aged burn mice, p <  0.05). By 24 hours, aged burn mice showed significantly higher expression (934.3 ±  76.75-fold) compared to young burn mice (711.1 ±  53.79-fold, p <  0.05). (Fig 2).

### Age-related differences in cytokine profiles

**Fig 3:**
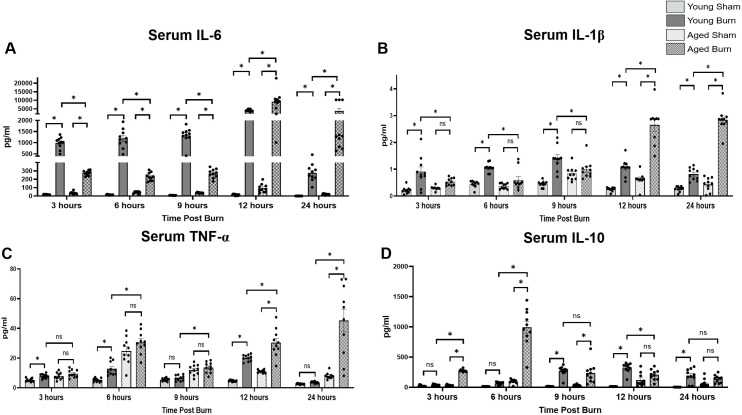
Comprehensive analysis of cytokine levels in the serum of young and aged mice at different timepoints post-burn. Cytokine serum concentrations (A) IL-6, (B) IL-1β, (C) TNF-α, and (D) IL-10 measured by multiplex pg/dl (±SEM). n = 10 per group. * p < 0.05, One-way ANOVA. Tukey’s multiple comparisons.

We analyzed key inflammatory mediators in serum to understand the molecular signals driving the hepatic acute phase response. IL-6, a principal activator of hepatic APP production, showed distinct age-dependent responses to burn injury [11]. IL-6 levels in young burn mice rose rapidly at 3 hours (990 ±  83.2 pg/ml), significantly higher than aged burn mice (274.6 ±  9.314 pg/ml, p <  0.05). This difference persisted at 6 hours (1187 ±  133.2 pg/ml in young vs. 232.1 ±  15.37 pg/ml in aged, p <  0.05) and 9 hours (1328 ±  115.0 pg/ml in young vs. 270.0 ±  16.77 pg/ml in aged, p <  0.05). However, at 12 hours, aged burn mice showed a dramatic increase (9124 ±  1922 pg/ml), significantly exceeding young burn mice (4131 ±  198.8 pg/ml, p <  0.05). This higher response in aged mice continued throughout 24 hours (3804 ±  1408 pg/ml vs. 276.3 ±  32.22 pg/ml in young, p <  0.05). (Fig 3A).

Interleukin-1β (IL-1β), a key mediator of the inflammatory response that stimulates APP production through NF-κB signaling [15], displayed similar age-specific patterns. Young burn mice showed higher early levels at 3 hours (0.908 ±  0.180 pg/ml) compared to aged burn mice (0.532 ±  0.043 pg/ml, p <  0.05). At 6 hours, young burn mice maintained higher levels (1.059 ±  0.052 pg/ml vs. 0.604 ±  0.123 pg/ml in aged, p <  0.05). However, by 12 hours, aged burn mice exhibited significantly higher levels (2.66 ±  0.241 pg/ml vs. 1.071 ±  0.099 pg/ml in young, p <  0.05), maintaining this elevation through 24 hours (2.85 ±  0.144 pg/ml vs. 0.821 ±  0.072 pg/ml in young, p <  0.05). (Fig 3B).

TNF-α, a pro-inflammatory cytokine that initiates the acute inflammatory cascade (13), showed distinct age-dependent responses. Young burn mice exhibited a significant early elevation at 3 hours (7.88 ±  0.465 pg/ml vs. 5.192 ±  0.352 pg/ml in young sham, p <  0.05) and 6 hours (12.82 ±  1.414 pg/ml), followed by an unexpected decrease at 9 hours (6.573 ±  0.600 pg/ml) before reaching peak levels at 12 hours (20.16 ±  0.686 pg/ml). Aged burn mice maintained consistently higher TNF-α levels than young burn mice throughout the study, but showed no significant elevation compared to aged sham controls until 12 hours (30.38 ±  2.925 pg/ml, p <  0.05 vs. aged sham), reaching maximum levels at 24 hours (45.26 ±  7.701 pg/ml, p <  0.05 vs. aged sham). At this final timepoint, young burn mice had returned to baseline (3.674 ±  0.188 pg/ml, p <  0.001 vs. aged burn). (Fig 3C).

IL-10, an anti-inflammatory cytokine that helps regulate and resolve the inflammatory response [17], exhibited early elevation in aged burn mice (275.4 ±  6.539 pg/ml at 3 hours), significantly exceeding young burn mice (38.47 ±  3.492 pg/ml, p <  0.05). This difference peaked at 6 hours (993.9 ±  99.41 pg/ml in aged vs. 67.69 ±  6.635 pg/ml in young, p <  0.05). Young burn mice showed peak IL-10 levels at 12 hours (323.3 ±  25.3 pg/ml), while aged burn mice maintained higher overall levels throughout the study period. (Fig 3D).

### STAT3 activation patterns

**Fig 4:**
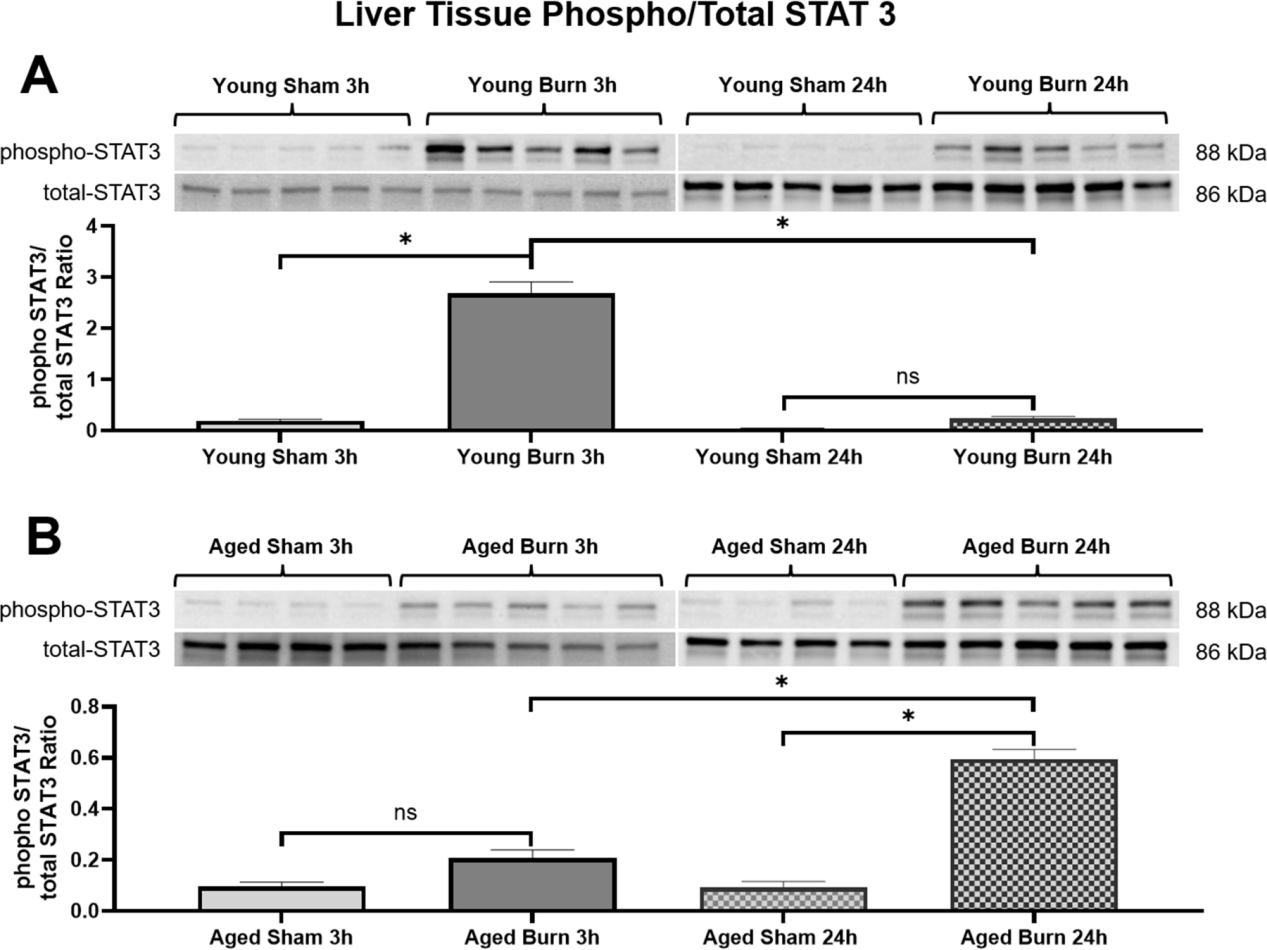
Western blot analysis of phosphorylated (p-STAT3) and total STAT3 (t-STAT3) levels in liver tissues of young (A) and (B) aged mice 3 and 24 hours postburn. Phosphorylated STAT3 was assessed and normalized to total STAT3. Shown are p-STAT3/t-STAT3 ratio (±SEM). n = 10 per group. * p < 0.05, One-way ANOVA. Tukey’s multiple comparisons.

STAT3, a critical transcription factor that mediates IL-6 signaling and regulates APP production in hepatocytes (34), showed significant age-dependent differences in activation. At 3 hours post-burn, young burn mice demonstrated markedly higher STAT3 activation (2.686 ±  0.226-fold increase vs. sham) compared to aged burn mice (0.208 ±  0.032-fold, p <  0.05). This pattern was reversed by 24 hours, with aged burn mice showing significantly higher STAT3 activation (0.596 ±  0.037-fold) compared to young burn mice (0.237 ±  0.041-fold, p <  0.05), consistent with the delayed inflammatory response observed in aged animals. (Fig 4).

## Discussion

Burn injuries induce a complex inflammatory response that involves multiple organ systems, with the liver playing a critical role in modulating inflammation through the acute phase response [4,8]. This study investigated the hepatic response to burn injury in young and aged mice, focusing on differences in cytokine dynamics and hepatic APP transcriptional and production. Our findings demonstrated a delayed hepatic inflammatory response in aged mice. This delay could be related to a downstream consequence of a hyporesponsive immune system in aged animals, as documented in other studies [7,35,36], which reduces the signaling needed for adequate liver activation. These results align with the broader understanding of the vulnerabilities faced by elderly burn victims and may help inform age-specific approaches to treatment.

Our findings show that aged mice exhibit a delayed increase of pro-inflammatory cytokines, such as IL-6 and TNF-α, in response to burn injuries. This early hypo-inflammatory state appears to disrupt the timeline of hepatic response, as the liver of aged mice only begins to activate and respond after this delay. Gomez et al. reported a similar age-related reduction in the acute phase response in aged rats, showing increased hepatic inflammation with a delayed cytokine response compared to younger rats [16]. Clinically, this phenomenon has also been documented in elderly burn patients, who exhibit reduced early cytokine levels and are associated with poorer outcomes [7]. Together, these studies underscore how aging impairs the prompt activation of immune signaling, leading to insufficient liver activation and the risk of a feedback loop that exacerbates systemic dysregulation [6,30,35].

The delayed rise of IL-6 in aged burn mice compared to younger counterparts in our study is noteworthy, as IL-6 is a primary activator of the acute phase response in the liver [11]. In young mice, IL-6 levels peaked early, at 3 hours post-burn, driving timely liver activation via STAT3 phosphorylation and subsequent APP production. In contrast, aged mice did not experience a robust IL-6 peak until 12 hours post-burn. This delay in cytokine signaling may hinder timely liver activation, as evidenced by the delayed STAT3 phosphorylation and APP synthesis we observed in aged mice. Consequently, this inadequate early immune signaling delays liver-driven inflammatory regulation, resulting in a feedback loop of immune dysregulation that can prolong inflammation [7,16]. Stanojcic et al. observed a similar delay in IL-6 production in elderly burn patients, which may result in prolonged systemic inflammation due to the liver’s impaired regulation of cytokines and APPs [35]. Interventions to enhance early immune signaling may help address this initial hypo-inflammatory state and improve outcomes in elderly patients.

The hepatic transcription of SAA1 and SAA2 genes differed in young and aged burn mice, with young mice exhibiting early increases in gene expression at 6-9 hours, while aged mice did not show significant transcriptional increases until 12 hours post-burn. The delay in transcription likely stems from the initial diminished presence of pro-inflammatory cytokines necessary for robust gene activation [11,32]. Thorn et al. noted that SAA gene transcription is highly sensitive to cytokine signals, with age-related immune function decline likely contributing to delayed transcription [32].

SAA, a liver-derived APP, modulates inflammation and facilitates tissue repair [14]. In young mice, SAA levels rose sharply at 12 hours post-burn, coinciding with early immune activation. However, aged mice demonstrated delayed and diminished SAA increases, probably due to the insufficient immune signaling that activates the liver. Abdullahi et al. have shown that lower SAA production in older patients correlates with poorer wound healing and increased risk of complications, underscoring the importance of timely SAA production in recovery [37]. The diminished SAA response in aged animals may, therefore, compromise immune regulation, further delaying the resolution of inflammation [7,32,33].

This study also highlights differences in STAT3 phosphorylation between young and aged mice. STAT3 is essential for transducing signals from IL-6 to initiate the acute phase response in hepatocytes [34]. Young mice exhibited rapid STAT3 activation as early as 3 hours post-burn, aligned with early IL-6 production. In contrast, aged mice showed delayed STAT3 phosphorylation at 24 hours post-burn. This delayed activation highlights how an initially insufficient immune response postpones liver signaling, which may, in turn, exacerbate systemic dysregulation. The reduced and delayed STAT3 activation in aged mice suggests impaired signaling pathways critical for the acute phase response. Alonzi et al. have highlighted the essential role of STAT3 in controlling the acute-phase response, which is compromised in our aged mouse model [34].

Our results relate to “inflammaging,” a chronic low-grade inflammation often seen in older individuals, heightening their susceptibility to exaggerated and prolonged responses following acute injuries [38]. The delayed and dysregulated inflammatory response observed here could reflect how aging impacts both baseline and acute inflammatory signaling, leaving elderly individuals vulnerable to ongoing inflammation and impaired recovery. Inflammaging likely contributes to the impaired signaling observed in aged animals, reducing the liver’s efficiency in producing rapid, coordinated responses to stress [39].

Regarding TNF-α dynamics, young mice showed an initial TNF-α spike followed by a decline at 9 hours and then another increase at 12 hours, likely to reflect a controlled feedback loop to regulate inflammation. Bode et al. noted that TNF-α can trigger anti-inflammatory mediators like IL-10, potentially explaining the 9-hour decline as part of this regulatory loop [11]. However, TNF-α levels remained elevated in aged mice without this regulatory drop. This may stem from the earlier hypo-inflammatory state and the delayed liver response, leading to a dysregulated feedback system that prolongs inflammation [6].

### Limitations and future directions

This study has several limitations. First, our use of healthy-aged mice may not fully reflect elderly patients with pre-existing comorbidities that could influence the inflammatory response to trauma. Future studies should examine the effects of common age-related conditions, such as diabetes or cardiovascular disease, on burn injury responses in aged animals. While our burn injury model is established, it may not encompass the full complexity of clinical scenarios. Research using diverse burn models or additional trauma would better simulate the clinical situations seen in elderly patients. Our focus on the first 24-hour post-injury limits insights into long-term effects. Extending observation periods to study inflammation resolution, tissue repair, and recovery could reveal further age-related differences in healing. Future research should also explore therapeutic interventions, such as early glucocorticoid administration or targeted cytokine modulation, to improve outcomes in aged animals. Investigating age-related effects on multiple organ systems, including immune, cardiovascular, and skin healing responses, would provide a more comprehensive understanding of post-burn responses. Additionally, while we analyzed hepatic responses, we did not directly measure liver damage in this study, which may affect interpretation. Previous work from our lab has shown that burn injuries can indeed induce hepatic damage in aged mice, which could further delay or compromise cytokine-driven liver activation [22,23]. Future studies should include liver injury markers to assess how hepatic integrity influences cytokine responses in aged animals. This study also used only female mice, based on previous findings on sex differences in burn outcomes; however, future work should include both sexes to understand interactions between age, sex, and burn trauma better. Addressing these limitations and pursuing these directions will improve understanding of age-related differences in burn injury responses, guiding age-specific treatments for elderly burn patients.

In conclusion, our study highlights critical age-dependent differences in the hepatic response to burn injury, underscoring the need for age-specific therapeutic strategies for elderly burn patients. These findings pave the way for future research to examine the extent of liver damage in aged burn victims and explore targeted interventions to improve outcomes in this vulnerable population.

## Supporting information

S1 File Raw data from ELISAs, PCRs, and western blot quantifications. This file contains the numerical results used to generate key findings of the manuscript, including inflammatory cytokines (IL-6, IL-1β, TNF-α, IL-10), acute phase reactants (SAA1, SAA2), and densitometry values for Western blot analysis across experimental groups and time points.
(PDF)

S1 Fig Raw Western blot images. This file contains uncropped Western blot images for phospho-STAT3 (P-STAT3) and total STAT3 (T-STAT3) from young and aged experimental groups at 3-hour and 24-hour time points, corresponding to the manuscript’s findings.
(PDF)
