# Correction: Delayed hepatic response and impaired cytokine dynamics in aged mice following burn injury: Implications for elderly patient care

**DOI:** 10.1371/journal.pone.0342189

**Published:** 2026-02-03

**Authors:** Israel Muro, Andrea C. Qualman, Kenneth Meza Monge, Akshay Pratap, Elizabeth J. Kovacs, Juan-Pablo Idrovo

There are errors in the Funding statement. The correct Funding statement is as follows: Supported by NIH institutes: K08GM134185 (JPI) R01GM153949 (JPI) R01AG018859 (EJK). The founders had no role in study design, data collection and analysis, decision to publish, or preparation of the manuscript.
