# Supplementary material for: Delayed hepatic response and impaired cytokine dynamics in aged mice following burn injury: Implications for elderly patient care
Source: PLoS One. 2025 Feb 24;20(2):e0316813. doi: 10.1371/journal.pone.0316813 (PMC11849828; doi:10.1371/journal.pone.0316813)
Supplement: S1 File — This file contains the numerical results used to generate key findings of the manuscript, including inflammatory cytokines (IL-6, IL-1β, TNF-α, IL-10), acute phase reactants (SAA1, SAA2), and densitometry values for Western blot analysis across experimental groups and time points. (PDF) [file pone.0316813.s001.pdf]

SAA

					Young Sham					
3 hours	0.957749	1.076991	1.046347	1.005462	0.913451	1.040149	0.968556	0.990269	0.979462	1.021756
6 hours	1.113705	0.97187	1.138582	1.170609	0.605235	0.916433	1.183985	1.061914	0.994329	0.84403
9 hours	0.850805	0.55464	0.611791	0.49576	0.619996	0.672591	0.794561	0.527631	0.649128	0.589203
12 hours	1.009561	1.028152	0.9591	0.836933	1.166254	1.072815	0.926237	0.886767	1.145699	0.968556
24 hours	1.07446	0.675566	0.35645	2.537073	0.35645	1.219925	0.794561	1.38654	1.549856	0.526295
					Young Burn					
3 hours	1.261014	1.066749	1.01229	1.042852	1.100806	1.096742	1.150397	1.054527	1.078919	1.103125
6 hours	5.014904	4.531771	5.752027	6.156224	4.955304	4.728616	5.281924	6.437318	4.895807	3.19852
9 hours	9.46653	8.712437	7.829226	8.607547	6.267829	9.859738	7.362316	8.195902	7.628381	9.061591
12 hours	23.41384	40.70556	30.29631	18.80352	35.05323	22.61936	28.44888	32.14384	25.26137	33.84163
24 hours	29.04599	52.59072	51.82206	33.82493	40.60541	37.16171	45.13915	48.67733	31.50131	44.3705
					Aged Sham					
3 hours	0.950754	1.035186	0.991404	1.022656	1.007533	0.97897	0.964498	1.049633	0.992436	1.007751
6 hours	0.70767	0.723015	0.933686	1.251199	1.38443	1.072815	0.886767	0.958454	0.814956	1.267309
9 hours	0.242704	2.206308	0.347595	1.97927	0.224124	1.038979	0.794561	1.265094	0.672591	1.219925
12 hours	1.364172	0.804316	0.918409	0.913103	1.32251	1.183985	1.061914	0.886767	1.219925	0.958454
24 hours	0.836881	1.017635	0.537093	1.608392	1.612444	1.122489	0.958454	1.38654	0.672591	1.265094
					Aged Burn					
3 hours	1.116411	0.922635	0.785098	0.988271	1.082027	1.030677	0.958454	1.072915	0.886767	0.945748
6 hours	1.159702	0.481407	0.693585	0.239087	1.229825	0.916433	0.794561	0.672591	1.038979	1.183985
9 hours	1.325481	2.290613	1.728665	3.277424	0.924233	0.325481	2.290613	1.728665	3.277424	1.224233
12 hours	45.99162	27.40303	36.54666	42.02879	48.61367	31.87526	38.3942	44.20446	35.69913	41.18126
24 hours	76.43075	69.4106	70.24048	74.1794	78.62179	72.97893	75.38988	71.77847	77.82126	73.5791

SAA1

					Young Sham					
3 hours	1.7568	0.734406	0.554003	0.9055	0.855959	0.821853	1.154633	0.922645	0.783695	1.052364
6 hours	2.245673	3.205431	1.025074	0.142827	0.948861	1.658394	2.174584	1.493828	1.203487	1.725138
9 hours	4.254669	9.598559	0.215837	1.384954	0.081916	3.546789	2.789654	4.123987	5.674322	6.321549
12 hours	0.919031	1.185769	0.470158	0.447522	1.196856	0.872346	1.023457	0.912346	0.745679	0.634568
24 hours	0.490462	9.219333	0.182947	22.09863	0.054702	6.543217	11.23457	8.456789	5.678912	10.34568
					Young Burn					
3 hours	5.599153	1.334451	3.604641	13.16857	13.10814	7.259256	8.123218	9.345148	10.45656	11.56651
6 hours	366.173	54.74486	46.78057	187.2428	150.2906	161.7593	142.5837	129.8746	178.9654	139.4877
9 hours	2758.64	8386.914	4278.246	11303.3	1020.318	5120.385	7894.124	6543.215	9378.456	7245.987
12 hours	5.393634	373.4827	1286.893	738.2326	555.9308	738.1235	895.4561	625.7893	812.3457	970.2346
24 hours	211.608	240.9301	241.2678	313.1054	402.7322	381.4123	512.8235	460.3568	439.5679	498.2346
					Aged Sham					
3 hours	0.303655	1.405371	2.343301	1.158646	0.931651	1.256479	1.673285	1.896558	1.456128	1.756515
6 hours	1.710975	0.03938	1.190185	2.147666	2.154666	1.448375	1.823496	1.305983	1.690375	1.521385
9 hours	0.275533	32.38384	0.934021	9.08122	0.013213	8.567431	10.23466	7.654322	11.78935	6.54322
12 hours	2.064914	0.38766	0.929289	1.344302	1.914575	1.523457	1.234568	1.678912	1.123457	1.345679
24 hours	1.839675	0.809949	0.422314	1.589153	2.461532	1.234568	1.876543	1.345679	2.123457	1.567891
					Aged Burn					
3 hours	2.310325	1.871557	0.07564	0.417631	1.24944	1.588543	2.128123	1.339124	1.894821	1.779874
6 hours	0.034238	0.191605	1.661518	0.004511	1.572564	0.734183	0.825174	0.912537	0.658393	0.741928
9 hours	0.82525	41.65999	27.03302	177.859	93.91872	67.23479	54.12346	38.98765	72.45679	85.32199
12 hours	984.9496	999.7592	724.6069	270.8622	997.5194	795.1235	867.2346	953.4568	812.3457	904.5679
24 hours	589.3592	281.8309	461.5635	255.1575	352.0575	727.4568	398.2346	341.5679	412.3457	379.1235

SAA2

					Young Sham					
3 hours	1.004702	0.90683	1.423112	0.841157	0.916897	1.21854	1.06187	0.971864	1.147405	0.782124
6 hours	1.354584	1.600737	0.902515	0.504572	1.012737	1.075029	1.214908	0.752953	1.354584	1.227423
9 hours	1.267506	1.764818	0.670935	1.1493	0.579744	1.086461	1.118721	0.942144	1.244244	1.194983
12 hours	2.012257	0.699266	4.19035	0.479017	0.354057	1.547651	1.492328	1.614872	2.183095	1.547651
24 hours	0.429228	4.452971	0.304455	13.24624	0.129732	2.578393	0.976543	5.156464	0.335442	1.294546
					Young Burn					
3 hours	3.775128	5.320348	5.605222	10.00436	6.80907	5.702586	6.861835	4.282916	2.822131	7.920083
6 hours	27.77401	16.88595	20.97305	32.5907	26.62334	25.96941	22.17353	29.57736	23.374	23.87376
9 hours	330.7261	481.1283	266.9359	474.5753	354.2397	345.5242	410.8536	280.1479	215.6315	393.1901
12 hours	298.6062	358.4186	303.3189	252.9477	253.4369	298.6062	293.3473	341.2644	276.1525	284.4738
24 hours	519.8824	930.4735	950.619	434.3642	747.6174	618.3743	724.5389	656.5917	656.5917	871.4957
					Aged Sham					
3 hours	0.547636	1.214573	1.503434	1.716553	1.678923	1.332304	0.940979	1.214573	1.514868	1.059322
6 hours	0.142557	1.742907	4.024734	1.833345	1.732896	1.895388	1.389256	2.401418	2.653439	1.137237
9 hours	0.297945	12.53248	0.667591	3.488472	0.114996	3.420097	5.772374	1.9797	3.420097	2.646107
12 hours	1.982744	0.552674	0.707333	1.290151	1.419678	1.190516	1.134248	1.038979	1.56143	1.265094
24 hours	1.352978	0.827979	0.527631	1.691839	2.111326	0.914726	1.285319	0.173983	1.798456	0.296135
					Aged Burn					
3 hours	1.551763	1.549856	0.1211	0.57942	1.110058	0.982439	0.860149	1.220971	0.738785	1.120415
6 hours	0.240027	1.284512	0.089901	0.830845	1.153357	0.603647	0.924939	0.353567	0.718278	1.019195
9 hours	17.45258	16.60905	14.14056	77.06177	43.19092	21.29098	39.64851	33.68994	27.24945	45.73333
12 hours	155.496	176.6138	193.1974	203.8328	159.5448	181.737	177.743	166.6492	188.5811	174.3033
24 hours	750.8652	1083.661	1079.138	773.4966	1466.188	649.1277	1012.385	849.5943	712.8364	962.721

IL-6

Sham Young Mice

3 hours	16.59	12.5	22.24	21.04	16.59	17	19.01	16.02	16.99	20.98
6 hours	16.03	22.75	9.24	8.83	7.07	14.456	13.789	17.234	18.567	15.678
9 hours	5.06	10.23	8.98	8.46	4.8	7.345	6.789	9.456	5.678	11.123
12 hours	25.33	3.47	22.42	0.35	3.09	15.789	18.456	20.123	12.678	17.345
24 hours	0	0.07654	0.065457	0.65464	0	0.096895	0.095465	0.068569	0.096869	0.196857

Burned Young Mice

3 hours	867	1363.34	493.83	642.99	1233.655	1098.456	1156.789	1032.345	987.654	1024.123
6 hours	1030.16	1938.87	483.78	736.39	1440.51	1275.456	1625.789	1150.234	980.567	1205.678
9 hours	1243.72	424.91	1829.64	1504.69	1219.19	1350.789	1401.234	1452.567	1303.89	1554.123
12 hours	3766.92	3862.7	4185.345	3918.78	4945.523	3864.365	4919.766	3016.476	3893.446	4937.169
24 hours	194	467	218	105	228	287.456	312.789	245.123	330.678	375.345

Sham Aged Mice

3 hours	35.28	18.62	26.72	72.98	31.6272	31.44	41.742	36.165	28.4596	42.46
6 hours	18.9	27.09	54.57	39.45	54.28	45.678	48.123	52.789	50.345	47.456
9 hours	29.72	33.40033	37.75	47.63	27.1	35.789	39.456	41.123	44.678	38.345
12 hours	46.2	32.82	198.69	48.3	83.45696	75.789	120.456	65.123	110.678	140.345
24 hours	7.26	14.62	31.8624	8.44	28.6552	21.345	19.789	24.456	22.123	18.678

Burned Aged Mice

3 hours	310.5036	314.63	227.32	237.9897	249.5859	295.123	284.456	265.789	275.123	285.456
6 hours	312.764	179.358	273.27	167.6455	184.6545	221.789	243.456	290.123	215.678	232.345
9 hours	211.6546	264.5645	175.4941	349.4432	291.1268	235.789	310.456	280.123	255.678	325.345
12 hours	22958.06	11647.68	2015.88	5366.86	1010.995	10246.62	9246.63	9371.256	8614.645	10761.66
24 hours	730.9334	631.7542	10246.65	1275.835	1356	10246.64	815.7725	10246.63	1193.143	1297.364

IL-1B

Sham Young Mice

3 hours	0.05	0.51	0.14	0.27	0.1	0.21	0.23	0.19	0.18	0.26
6 hours	0.56	0.36	0.55	0.14	0.41	0.52	0.47	0.53	0.48	0.44
9 hours	0.28	0.66	0.28	0.43	0.44	0.5	0.52	0.47	0.51	0.42
12 hours	0.18	0.26	0.3	0.08	0.31	0.27	0.29	0.24	0.32	0.3
24 hours	0.15369	0.295837	0.264645	0.068764	0.358694	0.246988	0.296787	0.264565	0.365499	0.35645

Burned Young Mice

3 hours	2.13	1.41	1.16	0.13	0.25	0.8	0.85	0.9	0.75	0.7
6 hours	1.3	0.82	1.03	1.32	0.82	1.06	1.05	1.04	1.07	1.08
9 hours	0.72	1.99	2.18	1.22	0.91	1.4	1.41	1.42	1.39	1.38
12 hours	0.69	1.2	1.22	1.71	0.54	1.07	1.05	1.06	1.08	1.09
24 hours	0.932456	1.149679	1.068583	1.005646	0.498879	0.556455	0.65968	0.749847	0.639855	0.945679

Sham Aged Mice

3 hours	0.45	0.26	0.14			0.28	0.29	0.3	0.31	0.32
6 hours	0.24	0.48252	0.25	0.31	0.426582	0.28	0.431545	0.483752	0.3	0.4922
9 hours	0.8	0.81	0.525202	0.64	1.42	0.97	0.96	0.67285	0.95	0.625404
12 hours	1.1	0.04	0.72	0.61		0.6	0.62	0.63	0.64	0.65
24 hours	0.65722	0.752654	0.521654	0.081355	0.465855	0.46856	0.698546	0.384568	0.025864	0.27865

Burned Aged Mice

3 hours	0.61	0.75	0.53	0.41	0.66	0.39	0.42	0.38	0.49	0.68
6 hours	0.217586	1.25	0.51953	0.43685	0.47256	0.315822	1.38	0.436218	0.512746	0.49584
9 hours	0.972241	0.64	0.735852	1.09	1.03	0.835	0.895144	1.89	0.762	1.12585
12 hours	1.49	2.2	1.87	3.98		2.88	2.89	2.9	2.87	2.86
24 hours	3	2.86565	2.694535	2.686945	2.76568	2.88	2.863645	1.958353	2.952554	3.83454

TNFa

Sham Young Mice

3 hours	4.57	6.08	4.33	4.76	4.18	4.9	5.5	6.7	3.8	7.1
6 hours	5.91	3.67	4.89	3.78	4	4.56	5.23	6.78	7.12	5.89
9 hours	5.79	6.38	4.72	5.95	4.77	5.1	6.45	4.35	7.2	3.9
12 hours	4.18	4.13	5.16	4.41	4.41	4.8	5.3	3.9	4.9	5.4
24 hours	2.65	2.04	2.55	2.81	2.48	2.7	3.15	2.35	2.9	2.6

Burned Young Mice

3 hours	8.58	8.46	7.613982	6.76	8.93751	7.25	9.15	10.3	5.5	6.25
6 hours	9.01	20.26	17.76	11.98	9.54	8.25	10.75	9.33	12.47	18.89
9 hours	4.15	4.96	6.92	8.6	9.15	5.25	7.45	6.1	4.3	8.85
12 hours	20.85	16.62	21.46	18.14	20.02	19.75	22.3	17.5	23.1	21.9
24 hours	3.66	4.29	3.9	2.69	3.65	4.12	3.15	4.55	3.78	2.95

Sham Aged Mice

3 hours	12.03	4.4	7.2	7.18324	6.510097	8.95	9.87	10.34	5.67	6.89
6 hours	17.64	8.32	21.9	38.48	35.05	22.15	26.78	19.34	30.56	28.47
9 hours	12.74	9.64	5.92	17.53	10.34	11.75	13.85	8.43	15.26	14.92
12 hours	10.73	9.74	9.71	11.7	12.617	10.85	11.32	10.25	12.45	11.15
24 hours	5.27	7.18	13.3	9.33		8.45	6.9	7.75	8.1	6.65

Burned Aged Mice

3 hours	5.78	7.03	8.85	11.16	12.35	11.89	7.45	13.78	8.34	8.56
6 hours	42.77	28.82	31.09	31.51	16.94	27.35	35.46	39.78	22.59	29.87
9 hours	16.30114	7.5	17.77	13.41	10.17	14.25	15.78	12.34	18.56	9.89
12 hours	47.52	32.23	14.73	30.5	26.3176	28.45	35.67	22.34	40.12	25.89
24 hours	12.5	73.172	8.04	46.1	68.5	35.75	57.8	23.65	72.9	54.2

IL-10

Sham Young Mice

3 hours	16.49	39.491	16.22	16.47	15.85	15.9	16.3	47.75	15.65	16.1
6 hours	16.49	13.47	14.73	11.58	9.18	12.85	15.32	10.75	14.25	13.9
9 hours	17.44	17.92	10.82	12.45	12.05	14.78	15.23	13.56	11.89	16.34
12 hours	13.79	12.53	13.59	13.18	8.83	12.25	14.34	11.78	13.67	10.89
24 hours	3.08	3.27	1.88	3.01	1.94	2.95	3.12	2.76	3.3	2.58

Burned Young Mice

3 hours	21.83	58.65	51.64	28.71	40.25	35.78	45.63	32.49	38.12	31.622
6 hours	39.78	24.27	81.15	93.38	77.64	68.45	72.3	64.15	85.5	70.25
9 hours	287.68	69.32	284.56	238.52	375.32	251.89	299.45	312.78	275.34	327.56
12 hours	390.83	363.39	127.62	293.6	403.06	315.78	358.45	277.34	372.89	329.56
24 hours	210.4	341.462	20	311.3	19.6	180.25	290.67	240.89	170.45	160.34

Sham Aged Mice

3 hours	19.61	28.17	18.11	41.104	52.71	31.25	29.45	35.67	26.89	32.78
6 hours	70.18	15.79	115.57	144.83	120.2	93.45	102.3	85.75	111.4	97.6
9 hours	27.89	40.47	23.82	14.9	60.87	33.45	28.78	37.56	41.23	34.89
12 hours	23.62	34.97	78.85	104.62	348.511	60.23	248.67	189.34	72.45	55.89
24 hours	15.2	12	51	226.3	72.61	28.45	35.78	22.56	140.89	18.34

Burned Aged Mice

3 hours	270.62	251.29	269.14	253.67	324.04	273.5	275.6	280.45	265.3	290.75
6 hours	914.76	1439.93	1239.92	848.68	328.19	1035.45	1120.3	955.75	1290.6	765.5
9 hours	638.32	100.62	298.46	85.25	52.73	235.89	312.45	158.67	275.34	198.76
12 hours	169.48	262.64	52.17	200.08	131.433	246.89	298.45	350.34	215.23	130.67
24 hours	61.37	148	76.2	57.5	158	200.45	145.78	100.34	250.89	170.56

Dens fig value

Young Sham 3h	Young Burn 3h	Young Sham 24h	Young Burn 24h
0.156416	3.389878	0.038028	0.205635
0.124273	2.788126	0.06713	0.365508
0.152557	2.119912	0.053277	0.227716
0.211244	2.856828	0.033704	0.116097
0.315098	2.277644	0.042798	0.270243

Aged Sham 3h	Aged Burn 3h	Aged Sham 24h	Aged Burn 24h
0.144663	0.295996	0.081031	0.613717
0.081716	0.268891	0.058749	0.640201
0.092086	0.13585	0.162485	0.452753
0.061611	0.183694	0.065346	0.613239
	0.152926		0.659298

young 3h

Lane	Band No.	Band Label	Mol. Wt. (KDa)	Relative Fro	Adj. Volume	Volume (Int	Abs. Quant	Rel. Quant.	Band %	Lane %
	1	1	N/A	0.147059	25840	74160	N/A	N/A	13.52596	6.834532
	2	1	N/A	0.147059	22425	62700	N/A	N/A	11.0536	6.881473
	3	1	N/A	0.154412	29120	68880	N/A	N/A	13.23636	7.586494
	4	1	N/A	0.147059	37303	82490	N/A	N/A	17.44027	10.1068
	5	1	N/A	0.139706	46656	77031	N/A	N/A	23.96007	11.39691
	6	1	N/A	0.161765	285743	433789	N/A	N/A	77.22033	38.53201
	7	1	N/A	0.161765	198785	299879	N/A	N/A	73.60172	34.12167
	8	1	N/A	0.169118	152233	236368	N/A	N/A	67.94781	31.63165
	9	1	N/A	0.183824	202332	249990	N/A	N/A	74.07196	33.53154
	10	1	N/A	0.183824	138335	189143	N/A	N/A	69.49028	29.88959
	1	2	N/A	0.772059	165200	252960	N/A	N/A	86.47404	43.69446
	2	2	N/A	0.801471	180450	310200	N/A	N/A	88.9464	55.37399
	3	2	N/A	0.779412	190880	298480	N/A	N/A	86.76364	49.72905
	4	2	N/A	0.764706	176587	263968	N/A	N/A	82.55973	47.84415
	5	2	N/A	0.779412	148068	241056	N/A	N/A	76.03993	36.16937
	6	2	N/A	0.794118	84293	140857	N/A	N/A	22.77968	11.36678
	7	2	N/A	0.808824	71297	122840	N/A	N/A	26.39828	12.23821
	8	2	N/A	0.830882	71811	148362	N/A	N/A	32.05219	14.92121
	9	2	N/A	0.830882	70824	143754	N/A	N/A	25.92804	11.73733
	10	2	N/A	0.830882	60736	117895	N/A	N/A	30.50972	13.12303

young 24h

Lane	Band No.	Band Label	Mol. Wt. (KDa)	Relative Fro	Adj. Volume	Volume (Int	Abs. Quant	Rel. Quant.	Band %	Lane %
	1	1	N/A	0.101695	7098	68874	N/A	N/A	3.663446	2.546879
	2	1	N/A	0.110169	10875	72150	N/A	N/A	6.290672	5.769996
	3	1	N/A	0.101695	8475	69075	N/A	N/A	5.058192	3.204765
	4	1	N/A	0.144068	7050	57750	N/A	N/A	3.260493	2.525524
	5	1	N/A	0.135593	7800	67800	N/A	N/A	4.104183	2.940345
	6	1	N/A	0.118644	47625	108825	N/A	N/A	17.05614	14.09232
	7	1	N/A	0.144068	85200	186150	N/A	N/A	26.7672	19.79439
	8	1	N/A	0.144068	105000	173475	N/A	N/A	18.54796	13.72684
	9	1	N/A	0.135593	48900	99225	N/A	N/A	10.40204	7.271916
	10	1	N/A	0.101695	40050	119400	N/A	N/A	21.2749	20.21196
	1	2	N/A	0.822034	186654	276120	N/A	N/A	96.33655	66.97453
	2	2	N/A	0.830508	162000	279000	N/A	N/A	93.70933	85.95304
	3	2	N/A	0.830508	159075	227325	N/A	N/A	94.94181	60.15315
	4	2	N/A	0.864407	209175	404925	N/A	N/A	96.73951	74.93283
	5	2	N/A	0.847458	182250	314100	N/A	N/A	95.89582	68.70229
	6	2	N/A	0.830508	231600	421725	N/A	N/A	82.94386	68.53085

7	2		N/A	0.855932	233100	477825	N/A	N/A	73.2328	54.15578
8	2		N/A	0.864407	461100	461100	N/A	N/A	81.45204	60.28042
9	2		N/A	0.838983	421200	421200	N/A	N/A	89.59796	62.63663
10	2		N/A	0.813559	148200	315225	N/A	N/A	78.7251	74.79182

Aged 3h

Lane	Band No.	Band Label	Mol. Wt. (KDa)	Relative Fr	Adj. Volume	Volume (Int	Abs. Quant	Rel. Quant.	Band %	Lane %
1	1		N/A	0.091603	18128	79728	N/A	N/A	12.63804	9.070894
2	1		N/A	0.10687	15747	77082	N/A	N/A	7.554257	3.943355
3	1		N/A	0.122137	15910	82302	N/A	N/A	8.432088	4.187415
4	1		N/A	0.129771	11310	69078	N/A	N/A	5.803571	2.977554
5	1		N/A	0.137405	53406	152306	N/A	N/A	22.83928	13.77857
6	1		N/A	0.129771	46580	141610	N/A	N/A	21.19103	13.01663
7	1		N/A	0.145038	60726	151380	N/A	N/A	26.38941	16.44675
8	1		N/A	0.152672	30295	97857	N/A	N/A	15.51871	7.685829
9	1		N/A	0.152672	49794	131064	N/A	N/A	42.95252	36.69202
1	2		N/A	0.770992	125312	369688	N/A	N/A	87.36196	62.70366
2	2		N/A	0.770992	192705	426909	N/A	N/A	92.44574	48.25708
3	2		N/A	0.778626	172774	396030	N/A	N/A	91.56791	45.47307
4	2		N/A	0.78626	183570	381930	N/A	N/A	94.19643	48.32799
5	2		N/A	0.763359	180428	446770	N/A	N/A	77.16072	46.54981
6	2		N/A	0.755725	173230	366180	N/A	N/A	78.80897	48.40855
7	2		N/A	0.78626	169389	355047	N/A	N/A	73.61059	45.87653
8	2		N/A	0.793893	164921	287097	N/A	N/A	84.48129	41.84039
9	2		N/A	0.793893	66134	313298	N/A	N/A	57.04748	48.73257

Aged 24h

Lane	Band No.	Band Label	Mol. Wt. (KDa)	Relative Fr	Adj. Volume	Volume (Int	Abs. Quant	Rel. Quant.	Band %	Lane %
1	1		N/A	0.124031	21648	71504	N/A	N/A	7.495741	5.049732
2	1		N/A	0.139535	11480	56580	N/A	N/A	5.54895	3.923767
3	1		N/A	0.132813	33456	86346	N/A	N/A	13.97739	9.158249
4	1		N/A	0.132813	12710	54694	N/A	N/A	6.133755	3.953073
5	1		N/A	0.140625	127674	275356	N/A	N/A	38.03127	25.41626
6	1		N/A	0.133858	143811	317463	N/A	N/A	39.03188	26.60979
7	1		N/A	0.133858	90364	211970	N/A	N/A	31.16516	21.18823
8	1		N/A	0.149606	120786	273880	N/A	N/A	38.0129	25.24422
9	1		N/A	0.134921	124722	273060	N/A	N/A	39.73354	25.4902
1	2		N/A	0.782946	267156	349156	N/A	N/A	92.50426	62.31829
2	2		N/A	0.79845	195406	281342	N/A	N/A	94.45105	66.78812
3	2		N/A	0.796875	205902	310780	N/A	N/A	86.02261	56.36364
4	2		N/A	0.804688	194504	290116	N/A	N/A	93.86625	60.49477
5	2		N/A	0.820313	208034	325048	N/A	N/A	61.96874	41.41365
6	2		N/A	0.818898	224634	339996	N/A	N/A	60.96812	41.56471
7	2		N/A	0.811024	199588	326934	N/A	N/A	68.83484	46.79869
8	2		N/A	0.834646	196964	350222	N/A	N/A	61.9871	41.16538
9	2		N/A	0.833333	189174	314634	N/A	N/A	60.26646	38.66265
