# Supplementary figures and images for: Delayed hepatic response and impaired cytokine dynamics in aged mice following burn injury: Implications for elderly patient care
Source: PLoS One. 2025 Feb 24;20(2):e0316813. doi: 10.1371/journal.pone.0316813 (PMC11849828; doi:10.1371/journal.pone.0316813)

P STAT3

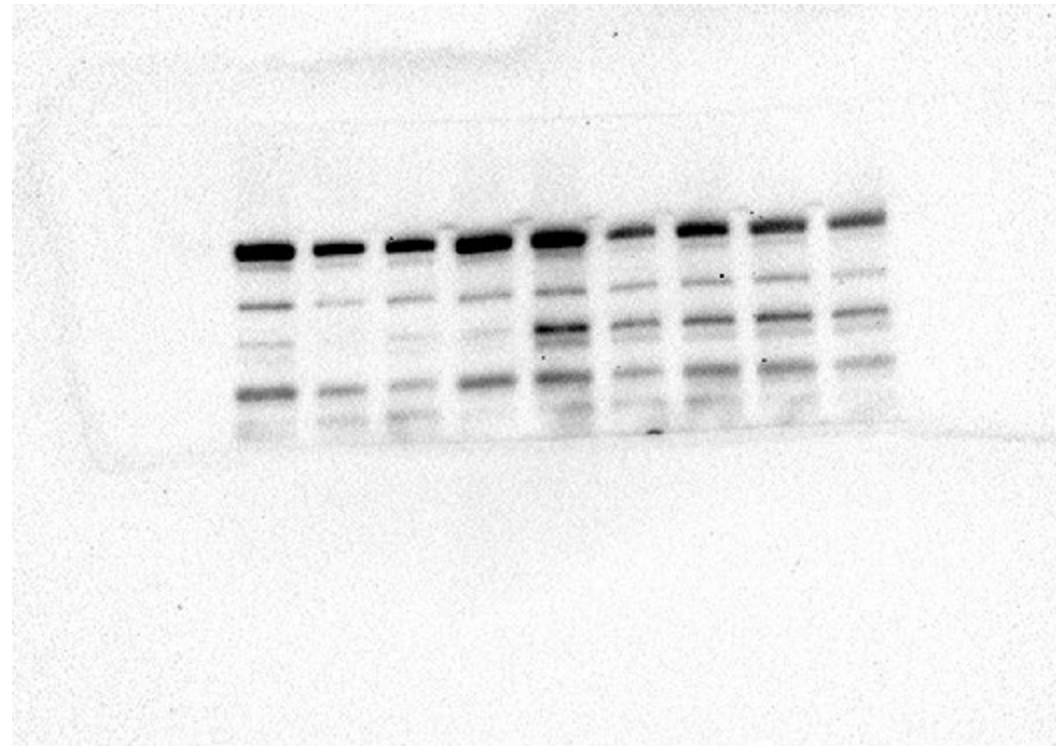

P STAT3

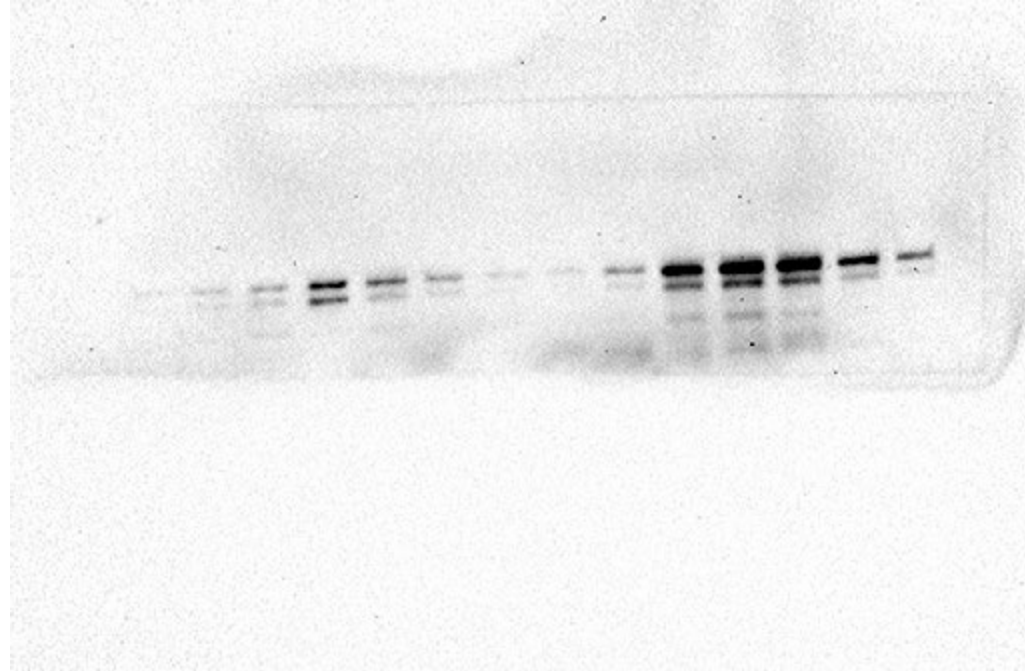

T STAT3

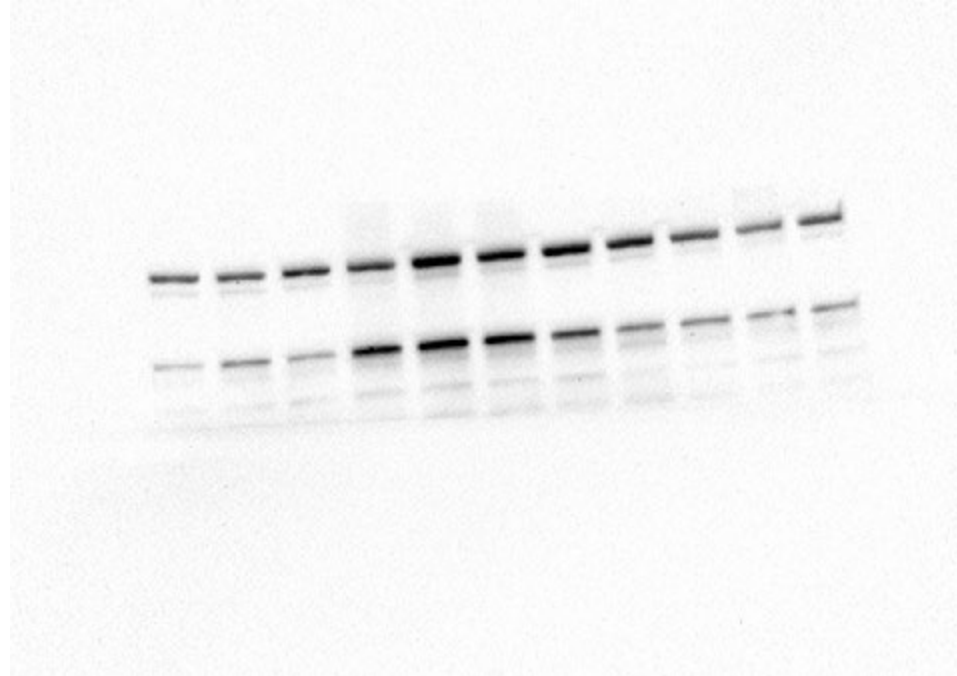

Aged 3h

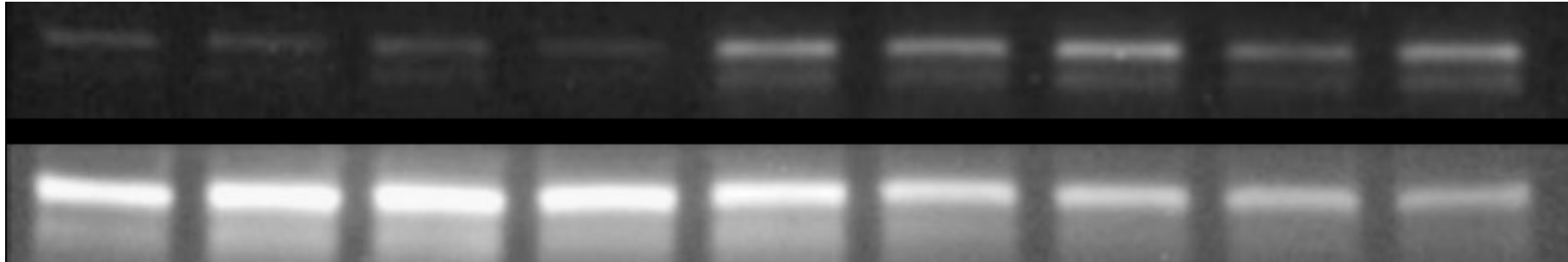

Aged 24h

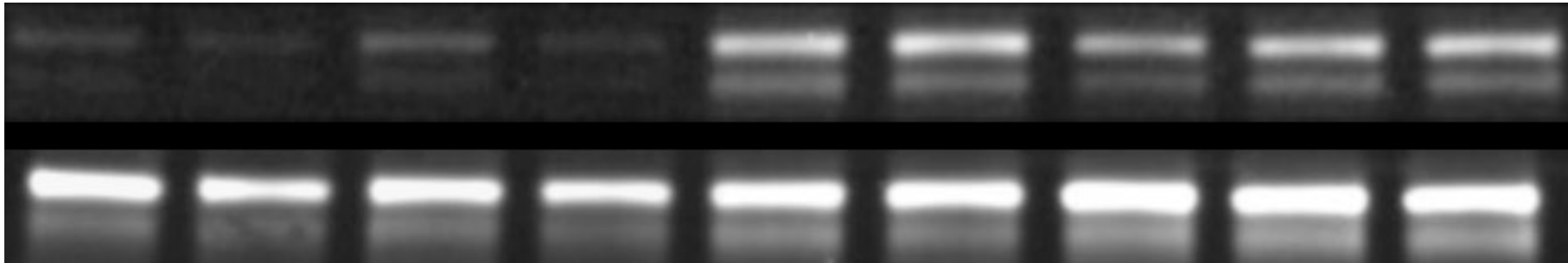

Young 3h

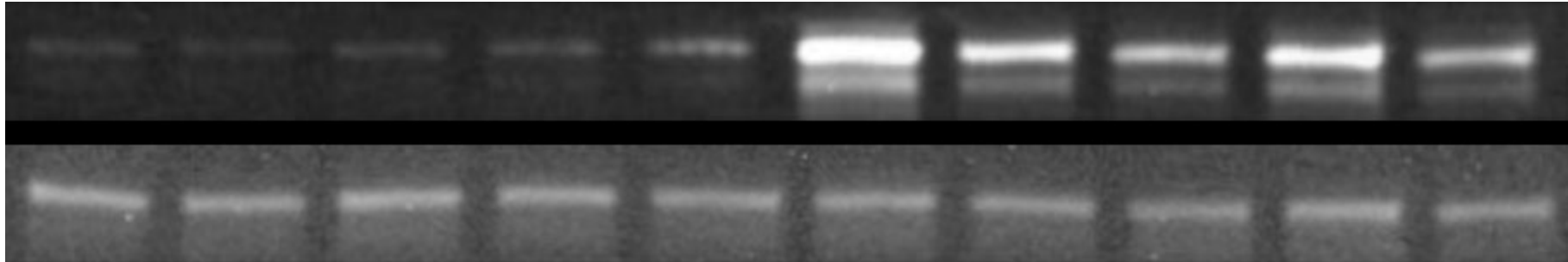

Young 24h

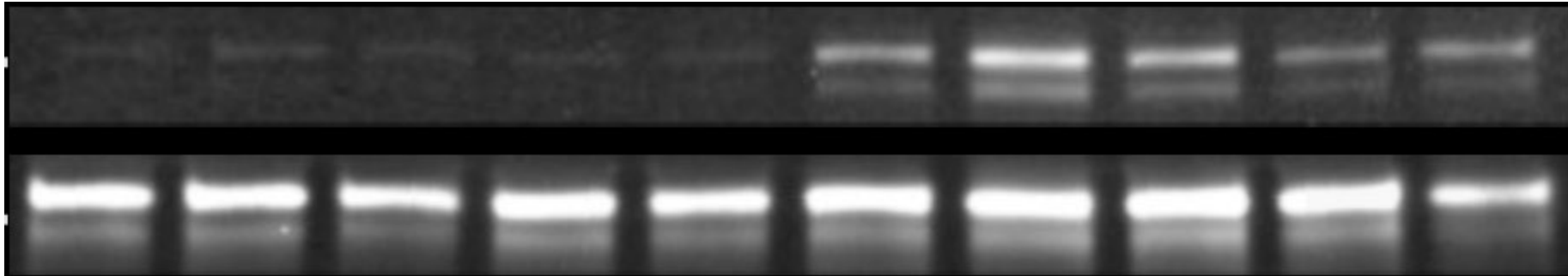

Supplement: S1 Fig — This file contains uncropped Western blot images for phospho-STAT3 (P-STAT3) and total STAT3 (T-STAT3) from young and aged experimental groups at 3-hour and 24-hour time points, corresponding to the manuscript’s findings. (PDF) [file pone.0316813.s002.pdf]
